# Bariatric Surgery Reduces Weight Loss, Comorbidities Prevalence, and Improves Quality of Life in the Southern Region of Saudi Arabia

**DOI:** 10.3390/medicina59101695

**Published:** 2023-09-22

**Authors:** Abdulaziz A. Arishi, Ibrahim Metaan Gosadi, Ibrahim Ali Hakami, Hussam Darraj, Faisal Abusageah, Khalid M. Hakami, Shaden A. Zaalah, Mohammed Awaf, Rawan Maghrabi, Afnan A. Alamer, Sulaiman Hamdi, Mohammad Abdu Jareebi, Amro M. Masmali, Ghalia H. Hakami, Weaam A. Najmi

**Affiliations:** 1 Department of Surgery, Faculty of Medicine, Jazan University, Jazan 45142, Saudi Arabia; 2 Department of Family and Community Medicine, Faculty of Medicine, Jazan University, Jazan 45142, Saudi Arabia; gossady@hotmail.com (I.M.G.);; 3 Faculty of Medicine, Jazan University, Jazan 45142, Saudi Arabiahakamikhaled5@gmail.com (K.M.H.);

**Keywords:** bariatric surgery, obesity, weight loss, metabolic surgery, sleeve gastrectomy, gastric bypass, diet, comorbidities, long-term outcomes, complications

## Abstract

*Background and Objectives*: Bariatric surgery has been proposed as a treatment option for type 2 diabetes, but there is limited research on its efficacy and the use of standardized outcome measures. Therefore, this study aimed to evaluate the efficacy of bariatric surgery in managing type 2 diabetes and to assess the BAROS protocol postoperatively. *Material and Methods*: This cross-sectional study was conducted in southern Saudi Arabia, involving 346 bariatric surgery patients aged 18–60. This study collected data through an electronic questionnaire distributed via a Telegram group and Twitter hashtag. Anthropometric data, postoperative complications, and the evolution of obesity-related comorbidities were collected. The quality of life was evaluated using the Moorehead–Ardelt questionnaire of the BAROS protocol. The total BAROS score was classified as “Insufficient”, “Moderate”, “Good”, “Very good”, or “Excellent”, considering the presence of comorbidities. The data were analyzed using SPSS software ver.23. *Results*: The mean age of the participants was 30.97 ± 8.49 years, and 70.81% were female. Sleeve Vertical Gastrectomy was the most common surgical technique used (*n* = 336). The excess weight loss percentage (EWL%) was 70.55 ± 22.57%, and 27.75% of participants achieved complete remission of type 2 diabetes. The total BAROS score was “Excellent” for 40.17% of participants and “Moderate” for 27.16%. The presence of comorbidities was negatively correlated with the BAROS score (r = −0.651, *p* < 0.001). *Conclusions*: Bariatric surgery effectively manages type 2 diabetes with a high rate of EWL% and complete remission. The BAROS protocol is a valuable tool for assessing the quality of life postoperatively, with most participants achieving a “Moderate” to “Excellent” score. Comorbidities negatively impact the BAROS score, highlighting the importance of managing these conditions postoperatively.

## 1. Introduction

Obesity and overweight have become a serious global public health concern. In the United States, obesity is a national epidemic affecting 41.9% of the population [1]. The same trend is reflected in European countries (Bulgaria, England, France, Germany, Greece, Ireland, Italy, Latvia, Poland, Portugal, Romania, and Spain), where about half of the population (48.1%) are overweight or obese (54.1% in men and 42.5% in women) and 12.6% are obese (11.3% in men and 13.8% in women) [2]. Several countries worldwide have also witnessed a doubling or tripling of the prevalence of obesity in the past three decades [2,3,4,5]. In 2014, the World Health Organization (WHO) estimated that about 1.9 billion adults were overweight and more than 600 million were obese, and projected that by 2030, more than 2.16 billion people will be overweight and 1.12 billion obese [6]. The prevalence is higher in Arabic countries, where Alqarni [7] estimates 52.9% of people in Saudi Arabia are obese, with a pronounced gender bias, where more females (67.5%) compared to males (38.2%) are overweight. The rising statistics indicate that obesity is an overt threat to public health around the globe, especially in Saudi Arabia, due to a considerably higher prevalence.

Obesity is abnormal or excessive fat accumulation, posing a health risk to the individual [5]. The WHO defines overweight as a body mass index (BMI) of 25.0 to 29.9 kg/m^2^, obesity as 30.0 to 39.9 kg/m^2^, and morbid obesity as 40.09 kg/m^2^ or higher [8]. Obesity occurs when dietary energy intake is substantially higher than energy expenditure. Thus, the principal causes of the obesity epidemic in Western nations are increased urbanization, sedentary lifestyles, and increased consumption of high-calorie processed food [8]. The most impacting factor is increased intake of processed food composed of high calories and fats but low fiber content. Poor meal planning (eating large portions of food or very frequently) leads to excessive calorie intake, which leads to fat accumulation in the body [8]. Similarly, in Arab countries, the dominant cause of obesity and overweight, among the highest rates in the world, is eating disorders. Melissa et al. [9] found a strong and positive correlation between eating disorders and higher BMI. Therefore, a combination of dietary habits and physical inactivity are the most significant contributors to the rising prevalence of obesity. 

Obesity and overweight are the leading predictors of a wide range of health conditions, decreased quality of life (QoL) and well-being. Excessive fat and an increase in waist circumference have been associated with an increased likelihood of developing a broad spectrum of non-communicable diseases (NCDs), such as type 2 diabetes mellitus, high blood pressure, cardiovascular diseases, especially hypertension, coronary artery disease, some types of cancers, and musculoskeletal disorders [10,11,12]. They are also associated with the pathophysiology of fatty liver disease and pregnancy-related complications [13,14,15,16]. Since NCDs are chronic health conditions, they increase the cost of healthcare and the risk of premature death, which places an economic burden on the individual, family healthcare sector, and the state [6]. Obesity is also associated with body image issues, leading to decreased self-esteem, social interaction, and work performance, which may lead to depression, stress, social isolation, and reduced QoL [17]. Thus, obesity has a deleterious effect on health, well-being, and economic stability, supporting the need for concerted efforts to reverse the increasing prevalence trend. 

Management of obesity focuses on lifestyle and medical interventions. Lifestyle modifications are the first-line treatment for helping obese and overweight individuals achieve and maintain a healthier weight through sustainable practices. Interventions such as having a balanced diet (reducing consumption of high-calorie food and overeating while increasing intake of organic and high-fiber foods) and regular physical activities reduce the mismatch between dietary intake and caloric body expenditure [18]. However, lifestyle interventions are inadequate to achieve significant weight loss for patients with morbid obesity (BMI ≥ 40.09 kg/m^2^), who may require complementary medical interventions [19,20]. Recommended medical treatment for morbidly obese individuals is a pharmacological treatment for weight loss maintenance, intragastric balloon (inflated in the stomach for six months to reduce the amount of food intake by promoting the feeling of satiation), and bariatric surgery, which targets to reduce the size of the stomach [21]. Therefore, effective management of obesity in morbidly obese individuals may require a combination of lifestyle and medical changes to the size and composition of the digestive system.

Bariatric surgery is the recommended treatment for individuals suffering from severe obesity complicated by comorbidities such as type 2 diabetes and hypertension because of its long-term positive effect on weight loss. The surgical procedure is usually considered when lifestyle changes, pharmacologic, and intragastric balloons have been ineffective in weight loss [21]. The four common types of bariatric surgery, (a) adjustable gastric banding, (b) Roux-en-Y gastric bypass, (c) sleeve gastrectomy, and (d) biliopancreatic diversion with a duodenal switch, are effective in achieving significant weight loss [10]. All four bariatric surgical procedures alter the digestive system to reduce food intake, leading to long-term excessive weight gain.

Gastric banding is a bariatric surgery that limits the amount of food in the stomach. The procedure involves the insertion of an inflatable silicone band around the upper part of the stomach to narrow its lumen, restrict food passage, and form a small proximal pouch of the stomach, which limits the amount of food intake weight and lessens comorbidities. The gastric band achieves a significant excessive weight loss percentage (EWL%) of up to 55% two years after the operation. The procedure also promotes remission of comorbidities such as diabetes (74%), hypertension (54%), dyslipidemia (40%) and sleep apnea (94%) [21]. However, the procedure has complications such as esophageal pouch dilatation, gastroesophageal reflux, band slippage, and erosion. Nevertheless, the complications are rare and rarely lead to loss of life. The procedure demonstrated the effectiveness of bariatric surgery in reducing excessive weight and remission of associated comorbidities.

Gastric bypass surgery involves reducing the stomach size by dividing it into upper and lower parts to limit food intake. The surgical procedure involves dividing the upper part of the stomach to form a proximal pouch, separating the intestines at the jejunal level, and attaching it to the new stomach pouch where ingested food passes [22]. Gastric bypass yields and maintains EWL% of up to 73% within 12 months and remission of diabetes (95%), dyslipidemia (80%), hypertension (81%), and sleep apnea (95%) [21]. Although the procedure has a lower mortality rate than open stomach surgery, it can lead to anastomotic leaks and internal bowel herniation that may lead to bowel obstruction and perforation; gastric bypass can achieve and maintain excessive weight loss for years and reduce the intensity of comorbidities. 

Sleeve gastrectomy exercises part of the stomach to reduce the size and quantity of food consumed. The procedure involves removing a large portion of the stomach (approximately 80%) to leave a narrow medial part (or sleeve). Decreasing the stomach size lowers motility and volume of ingested food passing, limiting caloric intake [22]. Sleeve gastrectomy achieves EWL% of up to 70% within 12 months and maintains for at least 36 months. The procedures also promote remission rates of diabetes (86%), hypertension (82%), dyslipidemia (83%), and sleep apnea (91%) [21]. The intervention has a low mortality rate, but its severe complication is leakage and gastroesophageal reflux. Sleeve gastrectomy achieves excessive weight loss by limiting food passing in the stomach and the number of calories the body absorbs.

Duodenal switch is an irreversible bariatric surgical procedure that involves sleeve gastrectomy and reducing the length of the small intestines to severely reduce the amount of food passing through the digestive system. The procedure occurs in two stages. The first step involves sleeve gastrectomy, exercising part of the stomach. The second phase is cutting the small intestines in two places and reattaching them to the duodenum and small intestines. Duodenal switch can lead to up to EWL% of 73% sustained for over eight years and remission of comorbidities such as diabetes [21]. However, common complications are perioperative anastomotic leaks, splenectomy, malnutrition, internal bowel herniation, and small bowel obstruction. Duodenal switch is an effective procedure for achieving and maintaining weight loss for years, although it is riskier regarding complications.

Bariatric surgery may be effective but can have severe physical and psychological implications. The procedure can lead to dumping syndrome, nutritional deficiencies, or psychological impact. Surgery alters stomach anatomy, which could lead to gastrointestinal complications and dumping syndrome. The condition results from gastric emptying, leading to gastrointestinal and vasomotor symptoms. Early dumping is more common after a meal and is characterized by abdominal symptoms, including pain, bloating, diarrhea, and borborygmi [21]. Patients can also experience late dumping 1 to 2 h after a meal due to reactive hypoglycemia, leading to systemic symptoms such as dizziness, fatigue, sweating, and general body weakness. The condition can lead to protein wasting, requiring dietary modification to manage the symptoms and maintaining adequate nutrition.

Bariatric surgery can also lead to nutritional deficiencies. The procedures alter nutritional intake and gastrointestinal absorption of micro- and macronutrients, especially in gastric bypass and duodenal switch that affect absorption. As a result, many patients require a lifelong balanced diet and nutrient supplement multivitamins and minerals such as folate, zinc, copper, and selenium, and iron, B12, calcium, and vitamin D. Some patients are advised to take fat-soluble vitamins that have undergone a duodenal switch procedure. The surgery can also lead to psychological impacts. Cases of harmful behaviors and the risk of suicide among patients who have undergone bariatric surgery are on the rise [21]. The underlying biological and behavioral mechanisms are unclear but may occur due to altered medication absorption and imbalances in peptides, hormones, and glucose. Others may develop postoperative eating disorders such as anorexia nervosa, bulimia nervosa, and binge eating, which result from altering eating habits. Therefore, patients who have undergone bariatric surgery should undergo nutritional and psychological counseling to improve outcomes on weight loss.

Bariatric surgery improves the quality of life associated with positive psychological feelings and the ability to achieve long-term excessive weight reduction and reduce stress associated with obesity. A meta-analysis review of 18 studies conducted between 2007 and 2021 with at least nine years of follow-up reports significant improvement in the psychical aspect of quality of life [11]. However, this study reports that health-related quality of life (HRQOL) increases significantly in the 1–2 years post-surgery and plateaus until 12 years but remains significantly higher than baseline [23]. In contrast, the authors report that improving the psychological aspect of quality of life is modestly possible because of psychological presuppositions. Thus, bariatric surgery promotes quality of life through positive feelings associated with bodyweight reduction and a positive perception of their physical image and health.

Existing study evidence supports that bariatric surgical procedures (gastric bypass, sleeve gastrectomy, gastric band, and duodenal switch) achieve and maintain long-term weight loss, high remission rates of comorbidities, and better QoL. Surgery reduces weight loss through restrictive (limiting food intake) and malabsorptive (altering calorie absorption in the stomach) mechanisms combined with dietary modifications, healthier lifestyles, and psychological and social support, leading to improved physical and mental health [24,25]. However, surgical procedures for weight loss maintenance are more effective in patients under the age of 50 years [26]. Bariatric surgery is now the most commonly used treatment for long-term weight loss, involving different types of gastrointestinal procedures such as Roux-en-Y gastric bypass, sleeve gastrectomy, duodenal switch, and jejunoileal bypass [27]. Therefore, for morbidly obese individuals with comorbidities, bariatric surgery is a safe and effective procedure to achieve long-term weight reduction, reduce comorbidities, and improve quality of life. 

Previous research has shown that bariatric surgery is primarily effective in modifying obesity-related nonalcoholic fatty liver disease (NAFLD) due to its impact on gastrointestinal (GI) hormones [28]. The procedure, which is the most effective long-term treatment for morbid obesity, leads to significant changes in gut hormones, playing a crucial role in promoting satiety, modulating energy homeostasis, controlling fat absorption, and influencing intestinal permeability. These hormonal changes are thought to contribute to the rapid improvement of obesity-related comorbidities, including NAFLD, often within days after surgery, even before significant weight loss occurs [28]. These findings underscore the importance of major peptides released by the enteroendocrine system, which influence satiety, energy regulation, fat absorption, and intestinal function. Additionally, they demonstrate that bariatric surgery not only results in sustained weight loss but also plays a role in the induction and long-term maintenance of weight loss through the modulation of gut hormones. Consequently, bariatric surgery is an effective approach to managing obesity by modifying the NAFLD implicated in the development of the chronic condition.

Obesity has been associated with an elevated risk of developing or exacerbating non-communicable diseases, psychological issues, decreased well-being and QoL. Although lifestyle-related interventions such as dieting and exercising can have long-term positive outcomes, they have few benefits for persons experiencing morbid obesity and comorbidities, which require medical intervention. However, most studies have been conducted in Western nations, whose lifestyle, culture, and medical approaches may differ from those of Arabic countries. Therefore, applying the findings to countries such as Saudi Arabia may yield different outcomes, leaving a study gap that the current study seeks to fill. Similarly, the present study aims to evaluate the efficacy of bariatric surgery in the management of type 2 diabetes, as well as to assess the BAROS (Bariatric Analysis and Reporting Outcome System) protocol postoperatively in the Saudi Arabian population.

## 2. Materials and Methods

### 2.1. Study Design and Participants

This study adopted the cross-sectional quantitative design to assess the quality of life, weight loss, and comorbidities in patients undergoing bariatric surgery in the southern region of Saudi Arabia. The method is beneficial in estimating the prevalence of health outcomes, understanding health determinants, and describing population features [26]. The design is relevant to the current study seeking to find the QoL, EWL%, and comorbidities remission among Saudis undergoing bariatric surgery. The subjects in the cross-sectional study are selected from an available population of potential relevance to the research purpose and aim. The design does not include prospective or retrospective follow-up because once the subjects are selected, the researchers collect the data and assess the associations between outcomes and exposures. Thus, a cross-section design helps study health outcomes among Saudi populations who are obese and diabetic and are undergoing bariatric surgery.

The study location is the southern region of Saudi Arabia, encompassing Najran, Abha, Jizan, and Bisha. This study involved 346 patients of both genders, aged between 18 and 60 years, who underwent Sleeve Vertical Gastrectomy (*n* = 336), Roux-en-Y Gastric Bypass (*n* = 7), or pancreaticoduodenal switch with duodenal switch (*n* = 3) between September 2022 and January 2023. This study excluded eight (8) participants who could not understand the study purpose and/or procedure (3), experienced debilitating complications (2), or declined to provide informed consent (3). The remaining 346 participated in this study and provided their responses to the survey questions distributed online. 

Data was collected through an electronic questionnaire distributed via a Telegram group and Twitter hashtag, including identification data (age, gender, date of surgery, education, profession, marital status, family income, and surgical technique), anthropometric data (weight, height, BMI, and overweight), and details on postoperative complications and the evolution of obesity-related comorbidities. Weight loss was assessed through the excess weight loss percentage (EWL%). Quality of life was evaluated using the Moorehead–Ardelt questionnaire of the BAROS protocol comprising five domains: self-esteem, physical activities, social relationships, work performance, and satisfaction levels. To obtain the total BAROS score, the QoL, EWL%, and comorbidities scores were summed, and the scores related to reoperation and primary and minor complications were subtracted. The final score was then classified as “Insufficient”, “Moderate”, “Good”, “Very good”, or “Excellent”, considering the presence of comorbidities.

### 2.2. Sample Size

The sample size was calculated using the EPI info program, considering a 95% confidence interval (CI), a 5% margin of error, and the total population of post-bariatric patients in Saudi Arabia. The estimated sample size was 300, adjusted to 346 to account for a 10% non-response rate. A pilot study comprising 10% of the required sample size (20 individuals) was conducted to assess the reliability and validity of the data collection survey. The pilot study’s results were used to make necessary improvements to the questionnaire. However, the findings should have been included in the final data analysis.

### 2.3. Statistical Analysis

The data were analyzed using the IBM SPSS^®^ application for Windows^®^ ver.23 (Statistical Product and Service Solutions, SPSS Inc., Chicago, IL, USA) in a 95% confidence interval, with statistical significance set at *p* < 0.05. Categorical variables were expressed using measures of central tendency as numbers (percentages) and numerical variables as mean and standard deviation (SD). The chi-square test was used to compare qualitative variables, while the t-test was employed to compare quantitative data. A chi-square test was conducted for category data, and a Pearson correlation was utilized to determine the correlation between the scale, ordinal variables, and various means of the question groups.

### 2.4. Ethical Approval

This study was conducted with ethical approval from the Scientific Research Ethics Committee (REC) of Jazan University (Reference number REC-44/02/294, date 13 December 2022). All participants provided informed consent before participating in the study.

## 3. Results

### 3.1. Baseline Characteristics

This study included 346 participants with a mean age of 30.97 ± 8.49 years. Among them, 29.19% were male, and 70.81% were female. Most participants had a Bachelor’s degree (65.61%), followed by high school education (26.30%). Abha (38.73%) was the most common residence of the participants. Most participants were Saudi (96.53%) and married (45.95%). Regarding income, 33.53% of participants had an income of 5000–9999 Saudi Riyal, and 24.57% had an income ≤4999 (Table 1).

### 3.2. Health-Related Characteristics

Table 2 presents the health-related characteristics of the sample. About 47.11% of the participants had chronic diseases, and the most common among them was difficulty conceiving (41.04%), followed by asthma (45.09%) and anemia (46.24%). Hypertension was present in 20.23% of the participants, while diabetes mellitus was found in 33.24%. Almost half of the participants (42.77%) had obstructive sleep apnea.

### 3.3. Bariatric-Related Characteristics

Participants’ mean body mass index (BMI) before surgery was 43.77 ± 6.46, which decreased significantly to 29.66 ± 6.88 after surgery. Hemoglobin A1C (HbA1C) also improved after surgery, with the mean before surgery being 9.08 ± 2.5 and after surgery being 5.74 ± 1.41. The mean diastolic blood pressure before surgery was 97.39 ± 12.41, which decreased to 85.31 ± 9.29 after surgery. Similarly, the mean systolic blood pressure before surgery was 156.89 ± 23.12, which decreased to 122.89 ± 15.3 after surgery. The rest of these characteristics are summarized in Table 3.

### 3.4. Complications Attributed to Bariatric Surgeries

Table 4 presents the complications of the bariatric surgery. Among the participants, 24.57% experienced malnutrition, while 39.60% had hair loss. Leakage occurred in 6.94% of participants, and sagging/slouching was present in 0.8671%. Only two (0.58%) participants experienced bleeding. The most common postoperative complication was no complication (49.71%).

### 3.5. Quality of Life among Participants after Bariatric Surgeries

After bariatric surgery, 82.95% of the participants reported feeling excellent, while 13.30% reported feeling good. The majority of participants reported improvement in physical activity (59.25%), social life (71.97%), and work (67.63%) after surgery. Only a few participants reported terrible outcomes in terms of satisfaction/feeling after surgery (1.16%), physical activity (4.05%), social life (2.31%), and work (2.89%). Table 5 summarizes the rest of the findings.

### 3.6. Health Parameters before and after Bariatric Surgery

**Figure 1:**
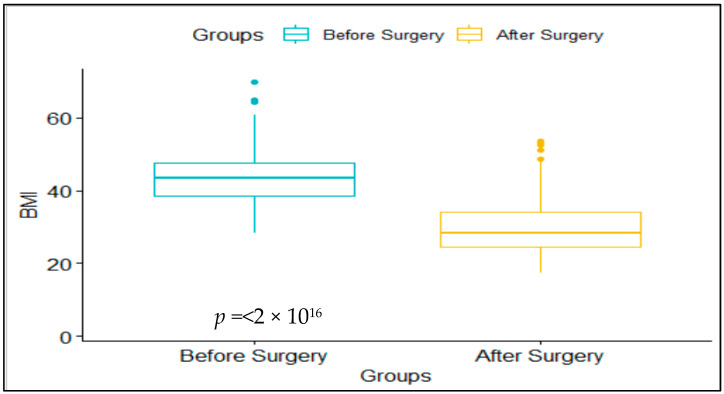
Body mass index (BMI): before and after bariatric surgery.

This study investigated the effects of bariatric surgery on several health parameters, including BMI, blood sugar, and blood pressure. The findings show significant improvements in all four parameters after bariatric surgery. Specifically, the mean BMI before surgery was 43.77 ± 6.46, while the mean BMI after surgery was 29.66 ± 6.88, with a significant difference of 14.12 (*p* < 0.001) (Figure 1). The mean HbA1c before surgery was 9.08 ± 2.5, while the mean HbA1c after surgery was 5.74 ± 1.41, with a significant difference of 3.36 (*p* < 0.001). The mean DBP before surgery was 97.39 ± 12.41, while the mean DBP after surgery was 85.31 ± 9.29, with a significant difference of 12.15 mmHg (*p* < 0.001). Finally, the mean SBP before surgery was 156.89 ± 23.12, while the mean SBP after surgery was 122.89 ± 15.3, with a significant difference of 34.00 mmHg (*p* < 0.001). These findings suggest that bariatric surgery can significantly impact a patient’s health, improving their BMI, blood sugar, and blood pressure levels.

## 4. Discussion

The increasing prevalence of obesity and overweight worldwide has become a significant public health issue due to its association with several comorbidities, such as hypertension, diabetes, hyperlipidemia, obstructive sleep apnea, coronary artery disease, osteoarthritis, fatty liver disease, and pregnancy-related complications [1,2,3,4,5,6]. The World Health Organization (WHO) defines obesity by BMI, the weight in kilograms divided by the square of height in meters [2]. Saudi Arabia has one of the highest prevalence of obesity globally (52.9%), with a higher rate among women than men [6]. However, Saudis are gradually recognizing the negative impact of obesity on health, well-being, and quality of life, influencing efforts to reduce body weight. Lifestyle modification is a tested and validated non-medical intervention to lose weight and maintain acceptable body fat. Strategies such as regular physical exercise and dietary changes are inexpensive, available to all, safe, effective, and recommended as the first-line interventions for obesity management [11,12]. However, these interventions may not be effective for patients with morbid obesity, who may require bariatric surgery to achieve significant weight loss. 

Diet has been identified as the most significant factor predicting the likelihood of developing obesity. Excessive body weight has been associated with an imbalance between caloric intake and the body’s needs or expenditure. According to Melisse et al. [9], eating disorders are the leading cause of comorbid obesity in the Arab World and are related to psychological effects such as depressive symptoms, anxiety, substance abuse, suicide attempts [4], and high mortality and relapse rates. Western literature associated poor feeding habits with Caucasian females due to their food culture [14]. The findings suggest a relationship between cultural factors and the likelihood of developing eating disorders. While the West perceives a thin body as ideal and achievable through diet and exercise, the traditional Arab notions of beauty are a curvy body that they associate with fertility and wealth. As a result, Arabs assumed eating disorders did not affect them until the late 1980s when the idea of the thin ideal began spreading in the Arab world. Therefore, due to Arab food culture and increasingly sedentary life, discipline to lifestyle interventions to manage weight gain remains a challenge to many. 

Medical interventions through surgery are the last-line intervention to achieve and maintain excessive weight loss for obese and comorbid individuals. In such individuals, lifestyle modification and medication may be inadequate to cause long-term weight reduction. Surgery can reduce weight and lead to better management of associated chronic illnesses instead of relying on mediation to treat a condition whose causative factors (obesity) still exist. Bariatric surgery is a surgical procedure that has effectively achieved substantial weight loss through limiting food intake and body absorption of nutrients with minimal complications. In addition to reducing body weight, surgery has complementary health benefits such as improved physical and mental health and QoL, mainly combined with dietary modifications, healthier lifestyles, and psychological and social support [14,15]. Thus, while dieting and excising may have had little effect on controlling obesity in Saudi Arabia, bariatric surgery represents an option for weight loss and management. 

The present study sought to evaluate the prevalence of health outcomes—quality of life, weight loss, and comorbidities—among obese individuals in Saudi Arabia with comorbid type 2 diabetes undergoing bariatric surgery. Other comorbidities evaluated are hypertension, obstructive sleep apnea, asthma, anemia, hypothyroidism, hypercholesterolemia, and joint pain. This study adopted the BAROS protocol postoperatively to evaluate the outcomes of interest from 346 patients who underwent sleeve vertical gastrectomy, Roux-en-Y gastric bypass, or pancreaticoduodenal switch with duodenal switch between September 2022 and January 2023. The results showed that bariatric surgery led to a significant reduction in weight and improvement in obesity-related comorbidities. The excess weight loss percentage (EWL%) was used to assess weight loss, and the Moorehead–Ardelt questionnaire of the BAROS protocol was used to evaluate QoL, comprising five domains: self-esteem, physical activities, social relationships, work performance, and satisfaction levels.

The study findings associate bariatric surgery with significant loss of weight and reduced remission of comorbidities. Pre- and post-intervention BMI decreased from 43.77 ± 6.46 to 29.66 ± 6.88 kg/m^2^. Based on the WHO classification of BMI, the surgical intervention led to an average weight loss that reclassified participants from morbid obesity to overweight within one year after surgery. Despite different bariatric surgical procedures, they all alter the digestive system’s anatomy, reducing average food intake and nutrient absorption. The surgical procedures create a smaller stomach pouch, narrowing food passage to the stomach or reducing food volume consumed during a meal. The changes alter hormonal production, which regulates appetite and metabolism. For instance, altering stomach anatomy interferes with the secretion of ghrelin hormone (responsible for stimulating the feeling of hunger) to reduce appetite or stimulate the production of GLP-1 and PYY hormones to create a feeling of fullness and satiety. Therefore, bariatric surgery achieves weight loss by simulating maladaptive hormonal processes to create a feeling of satiation, reducing intestinal nutrient absorption, limiting food intake, and altering feeding habits. 

Despite the short-term success of bariatric surgery in causing significant weight loss, psychopathological factors such as eating behaviors are necessary to maintain a successful weight maintenance program. About a third of patients who have undergone bariatric surgery gain suboptimal weight loss or significant gain within the first five postoperative years. Although the present study indicates significant weight loss, the short period is insufficient to determine whether participants can maintain the desired body weight. According to Saccaro [29], disordered eating behavior, such as emotional eating, can reverse the effects of dietary recommendations and surgical interventions in the long term, which are critical to maintaining weight loss and adherence to a healthy post-surgical diet. Despite bariatric surgical procedures limiting food intake and leasing to maladaptive nutrient absorption in the stomach and the more extensive digestive system, continued dieting is crucial to the maintenance of weight loss. As a result, it is necessary for bariatric surgeons to consider and characterize patterns of disordered (or pathological) eating styles and psychiatric symptoms before bariatric surgery. Therefore, recognizing gender differences in pathophysiological eating disorders helps determine recommendations for pre- and post-surgical processes to increase the success of the process in achieving and maintaining weight loss in the long term. 

Bariatric surgery can also lead to nutritional deficiency, which can affect the effectiveness and outcome of the weight loss program. Patients undergoing gastric banding and duodenal switch procedures can suffer from nutritional deficiency because they affect how the stomach and small intestines absorb micronutrients [21]. They may require additional treatment, such as a balanced diet and multivitamins for life. The impact on nutrients can also lead to altered medication absorption and imbalances in peptides, hormones, and glucose, leading to mental health issues. Postoperative eating disorders such as anorexia nervosa and bulimia nervosa may also affect the success of the surgery. Patients who have undergone bariatric surgery require nutritional and psychological counseling to ensure the program’s long-term success. Therefore, future studies should examine the impact of psychological barriers on the success of bariatric surgery. 

The present study findings suggest an association between bariatric surgical procedures and comorbidities. Although the present study’s period was short to allow assessment of comorbidities during pre- and post-intervention periods, participants blood pressure—diastolic and systolic blood pressure (BP)—provided a surrogate measure of hypertension, the leading comorbidity in morbidly obese patients. The decrease of both diastolic and systolic BP from 97.39 ± 12.41 to 85.31 ± 9.29 mm Hg and 156.89 ± 23.12 to 122.89 ± 15.3 mm Hg, respectively, indicates that bariatric surgery reduces hypertension. Since blood pressure is elevated secondary to cholesterol plaque buildup in the coronary arteries in obese patients, the surgery causes a reduction in body and coronary artery fatty deposits to cause a decrease in blood pressure. The findings reflect those of previous studies reporting gastric bypass reduces diabetes, dyslipidemia, hypertension, and sleep apnea remission by 95%, 80%, 81%, and 95%, respectively. Sleeve gastrectomy reduces diabetes, hypertension, dyslipidemia, and sleep apnea by 86%, 82%, 83%, and 91%, respectively [10]. The current findings are consistent with the literature review that bariatric surgery limits food intake, creates a feeling of satiation, and causes maladaptive stomach nutrient absorption to cause substituent reduction in body fatty deposits.

Previous studies support current findings associating surgical procedures with reducing body weight and improving mental health. Excessive body weight may lead individuals to suffer from sleep apnea and poor sleep quality. Excessive weight loss after surgery can alleviate these sleep-related issues, leading to better sleep and overall restfulness. The surgical intervention also improves clinical management of obesity-related comorbidities, such as type 2 diabetes, hypertension, and obstructive sleep apnea [19,20,21]. A systematic review and meta-analysis of 11 studies involving 796 patients with type 2 diabetes who underwent bariatric surgery showed that it led to remission of diabetes in 60% of patients, with sustained remission rates of up to five years postoperatively [22]. Although the present study did not directly evaluate the effect of comorbidities and their association with mental health, a reduction in associated diseases leads to a perception of a better life and reduced stress and anxiety.

The present study also reports that bariatric surgical interventions promote health, well-being, and QoL. The BAROS score is a tested and validated measure of QoL scoring on self-esteem, physical activities, social relationships, work performance, and satisfaction levels while considering adverse health outcomes such as reoperation of major and minor complications. The surveyed participants had an improved total postoperative BAROS score, indicating a better QoL, consistent with previous studies [14,15]. In particular, the findings indicated that most patients (80.3%) reported good, very good, or excellent outcomes, according to the BAROS classification. This study also found that sleeve vertical gastrectomy was the most commonly used procedure, consistent with previous studies showing its safety and effectiveness in reducing weight and improving comorbidities [30,31]. Bariatric surgical procedures improve the quality of life physically and psychologically [8,32,33,34]. Excessive reduction of body fat leads to the experience of improved mobility and the ability to engage in physical activities they could not participate in before surgery, leading to a more active and fulfilling lifestyle. Thus, bariatric surgery improves QoL by causing patients to experience improved metabolic health, resulting in higher energy levels and reduced fatigue, allowing them to enjoy daily activities more fully.

The present study also finds that bariatric surgery increases remission of comorbidities, which exacerbate adverse health outcomes and improve health outcomes and physical and emotional well-being. The benefits of bariatric surgery are not limited to weight loss and comorbidity management [33]. The present findings suggest that post-surgery, there is excellent satisfaction (83%), physical activities (59%), social life (72%), work-life (67%), and associated diseases (54%). Improvement in body image and self-esteem that often accompanies weight loss can contribute to enhanced mental health and psychological well-being. As patients lose weight and gain confidence, they may experience improved social interactions and emotional well-being because of a reduction in stress previously associated with social isolation and difficulties in personal relationships; the findings are consistent with previous studies that report patients who undergo bariatric surgery may also experience improved mental health outcomes, such as reduced symptoms of depression and anxiety [35]. In addition, bariatric surgery may improve social functioning and quality of life, including increased participation in physical and leisure activities [36,37]. Thus, excessive weight gain reduction leads to positive psychological outcomes associated with increased socialization and reduced stress associated with body image, suggesting improvement in physical and mental well-being.

The success of bariatric surgery in achieving excessive weight for morbidly obese patients has been supported by the present study findings and the literature. However, incidents of minor and adverse complications have been associated with the procedure, which healthcare professionals and patients should be aware of before consenting to surgery. The current study finds complications were joint post-bariatric surgery despite achieving the clinical endpoint of weight loss and high comorbidities remission rates. Among the 346 participants, about a quarter (24.57%) experienced malnutrition, and more than a third (39.6%) had hair loss. Few others had leakage (6.9%), sagging/slouching (0.9%), and bleeding (0.6%). However, about half (49.7%) reported no complications, and no fatal cases were reported in all the 346 participants. The findings suggest that although bariatric surgery is relatively safe, it can lead to minor complications for many patients, which supports the need to create awareness for the patient before consenting to surgical operations.

The present findings are consistent with the literature reporting minor and adverse complications. Although fatal cases are rare, complications are costly and associated with a negative perception of a repeat procedure, leading to stress and anxiety for affected patients. Gastric bypass has a significantly lower 30-day mortality rate (0.2%) than open stomach surgery (2.1%). However, the procedure could lead to serious complications such as anastomotic leaks (3–5%) and internal bowel herniation (3.1%), potentially leading to bowel obstruction and perforation. Sleeve gastrectomy had a meager mortality rate, but leakage in 2–3% of the patients portends a serious complication. Other complications are strictures (4%) and gastroesophageal reflux. Finally, the duodenal switch has a meager mortality rate (<1%). Still, it could lead to complications such as perioperative anastomotic leaks (3–4%) and splenectomy (<1%), and later malnutrition (4%), internal bowel herniation, and small bowel obstruction (2–7%) [21]. The risk of developing complications related to bariatric surgery is higher among the aged. Still, a lack of studies undermines gaining such knowledge through published books, online libraries, and security.

Patients’ knowledge of significant complications is necessary to select the most appropriate and safest bariatric surgery option to increase reversibility. One approach to dealing with complications is revising or reversing the surgery to prevent adverse side effects and their impact on health and well-being. Gastric band revision or removal is relatively simple and commonly associated with high band failure or intolerance rates, leading to nausea, dysphagia, and failure. Gastric bypass is also reversible to result in the resolution of complications and their symptoms, but cases of reversal have not been reported or are rare [21]. Sleeve gastrectomy is irreversible, and revision or reversal may be possible, but it carries considerable risk to the health and life of the patient. Since the reversal of bariatric surgical procedures is an effective way to treat complications, it is vital to inform patients of potential side effects and complications before the surgery to allow them to make informed and objective choices of the safest approach. 

Bariatric surgery has demonstrated safety and efficacy in significantly reducing weight and associated benefits such as reduced risk of developing or exacerbating commodities and improved QoL. The procedure is the last intervention to achieve immediate and long-term excessive weight loss when other strategies such as lifestyle modification and meditation have achieved less than adequate results. However, bariatric surgery is not safe and productive for all morbidly obese patients. The decision to undergo bariatric surgery should consider the risk of major and minor complications that may affect medical effectiveness. The choice should depend on carefully considering the patient’s circumstances, including their BMI, comorbidities, and overall health status. Patients considering bariatric surgery should be aware of the potential risks and complications associated with the procedure, such as bleeding, infection, and nutrient deficiencies that can lead to adverse health outcomes.

This study has several limitations that should be considered when interpreting the findings or applying them to a different but related population of diabetic and obese people who have undergone bariatric surgery. The cross-sectional design adopted had one major limitation. The method does not allow for the conclusion of causality because it is non-randomized, and the directionality between study variables cannot always be determined. The relationship between variables and determining the predictor versus outcome variable can present inaccuracies and sometimes need to be more credible. In addition, the strategy to gather data using self-reported questionnaires and through social media platforms raises issues of recall and social desirability biases. Using online channels to collect participant data complicates the researchers’ ability to achieve comprehension and confirm participant identity. Thus, the lack of causality and ability to confirm participant authenticity can affect the accuracy and reliability of the response and, ultimately, findings. 

Another limitation is that this study included patients who had undergone three different types of bariatric surgery (gastric bypass, sleeve gastrectomy, and duodenal switch), each with different levels of safety and efficacy on weight loss and remission rate of comorbidities. However, data analysis did not conduct or adjust for multiple comparisons of BMI, surgical types, and comorbidities. As a result, the findings could not determine the impact of weight loss on morbid obese, obese, and overweight patients, the safest and most effective bariatric surgical procedures, and the effect on comorbidities remission within the pre- and post-study period. Finally, when evaluating complications related to bariatric surgery and quality of life post-operations, the analysis did not adjust for potential confounders such as demographic characteristics such as gender and age, which could impact the findings. These limitations warrant further study on how age and sex may affect complications and QoL after bariatric surgery. Finally, the study did not evaluate long-term outcomes or assess the effect of the different surgical procedures on weight loss and comorbidities. A longitudinal study with a larger sample size and more extended follow-up periods is needed to address these limitations.

Morbid obesity is a medical condition that increases the risk of developing chronic health conditions affecting social, physical, and psychological health and well-being. Traditional exercise and diet approaches can be practical for overweight and obese individuals but less effective for morbidly obese individuals who require medical interventions. Bariatric surgery is a medical procedure that alters the anatomy of the stomach and digestive systems to limit food intake and lead to malabsorption of nutrients, leading to significant weight loss. Secondary outcomes of excessive weight loss include reducing the risk of developing or exacerbating comorbidities such as diabetes, hypertension, and obstructive sleep apnea. Bariatric surgical procedures are safe and effective in improving the physical and mental health and well-being of morbidly obese individuals. The procedures cause excessive weight loss within a year. They are maintained for a long time, leading to a reduction in psychological distress associated with body image, effective management of comorbidities, and improvement of QoL. Still, patients should be aware of potential complications to allow them to make objective risk-benefit analyses before consenting to surgical operations. However, the current findings are short-term, within a year, warranting further studies to evaluate long-term outcomes and the effect of age, sex, and presence of comorbidities on surgery, health and well-being. Therefore, healthcare professionals should work closely with patients to determine the most appropriate treatment plan for their needs and circumstances.

## 5. Conclusions

Bariatric surgery effectively manages type 2 diabetes with a high rate of EWL% and complete remission. The BAROS protocol is a valuable tool for assessing the quality of life postoperatively, with most participants achieving a “Moderate” to “Excellent” score. Comorbidities negatively impact the BAROS score, highlighting the importance of managing these conditions postoperatively.

## Figures and Tables

**Table 1:** Baseline characteristics of the sample (*n* = 346).

Characteristics	Mean ± SD
Age	30.97 ± 8.49
Characteristics	Frequency (%)
Gender	
Male	101 (29.19%)
Female	245 (70.81%)
Education	
Intermediate and below	13 (3.76%)
High school	91 (26.30%)
Bachelor	227 (65.61%)
Postgrads	15 (4.34%)
Residence	
Abha	134 (38.73%)
Beishah	27 (7.80%)
Jazan	160 (46.24%)
Najran	25 (7.23%)
Income (Saudi Riyal)	
“≤4999”	85 (24.57%)
“5000–9999”	116 (33.53%)
“10,000–14,999”	79 (22.83%)
“≥15,000”	66 (19.08%)
Nationality	
Saudi	334 (96.53%)
Non-Saudi	12 (3.47%)
Marital status	
Single	169 (48.84%)
Married	159 (45.95%)
Divorced/widowed/widower	18 (5.20%)

Abbreviations: SD: Standard deviation, *n*: Sample size.

**Table 2:** Health-related characteristics of the sample (*n* = 346).

Characteristics	Frequency (%)
Chronic diseases	
Yes	163 (47.11%)
No	183 (52.89%)
Hypertension	
Yes	70 (20.23%)
No	276 (79.77%)
Diabetes Mellitus	
Yes	115 (33.24%)
No	231 (66.76%)
Obstructive sleep apnea	
Yes	148 (42.77%)
No	198 (57.23%)
Difficulty to conceive	
Yes	142 (41.04%)
No	204 (58.96%)
Asthma	
Yes	156 (45.09%)
No	190 (54.91%)
Anemia	
Yes	160 (46.24%)
No	186 (53.76%)
Hypothyroidism	
Yes	155 (44.80%)
No	191 (55.20%)
Hypercholesterolemia	
Yes	157 (45.38%)
No	189 (54.62%)
Joint pain	
Yes	159 (45.95%)
No	187 (54.05%)

Abbreviations: *n*: Sample size.

**Table 3:** Bariatric-related characteristics of the sample (*n* = 346).

Characteristics	Mean ± SD
Body mass index (BMI)	
Before surgery	43.77 ± 6.46
After surgery	29.66 ± 6.88
Hemoglobin A1C (HbA1C) *	
Before surgery	9.08 ± 2.5
After surgery	5.74 ± 1.41
Diastolic blood pressure **	
Before surgery	97.39 ± 12.41
After surgery	85.31 ± 9.29
Systolic blood pressure **	
Before surgery	156.89 ± 23.12
After surgery	122.89 ± 15.3

*n*: Sample size; * *n* = 86, ** *n* = 103. SD: Standard deviation.

**Table 4:** Bariatric-related complications (*n* = 346).

Characteristics	Frequency (%)
Malnutrition	85 (24.57%)
Hair loss	137 (39.60%)
Leakage	24 (6.94%)
Sagging/slouch	3 (0.8671%
Bleeding	2 (0.58%)
Light-headed/dizziness	7 (2.02%)
Cholecystitis/gallstones	5 (1.45%)
No complication	172 (49.71%)

Abbreviations: SD: Standard deviation, *n*: Sample size.

**Table 5:** Quality of life after bariatric surgeries (*n* = 346).

Characteristics	Frequency (%)
Satisfaction/feeling after surgery	
Excellent	287 (82.95%)
Good	46 (13.30%)
No change	9 (2.60%)
Bad	4 (1.16%)
Physical activity after surgery	
Excellent	205 (59.25%)
Good	104 (30.06%)
No change	23 (6.65%)
Bad	14 (4.05%)
Social life after surgery	
Excellent	249 (71.97%)
Good	75 (21.68%)
No change	14 (4.05%)
Bad	8 (2.31%)
Work after surgery	
Excellent	234 (67.63%)
Good	82 (23.70%)
No change	20 (5.78%)
Bad	10 (2.89%)
Associated diseases after surgery	
Slight improvement	186 (53.76%)
No change	21 (6.07%)
Worse	4 (1.16%)
No associated diseases	135 (39.02%)

Abbreviations: *n*: Sample size.

## Data Availability

The data presented in this study are available on request from the corresponding author.

## List of Abbreviations

BAROS	Bariatric analysis and reporting outcome system
EWL%	Excess weight loss percentage
HRQOL	Health-related quality of life
LABS-2	Longitudinal assessment of bariatric surgery
LRYGB	Laparoscopic Roux-en-Y gastric bypass
LSG	Laparoscopic sleeve gastrectomy
QoL	Quality of life
RYGB	Roux-en-Y gastric bypass
T2DM	Type 2 diabetes mellitus
WHO	World Health Organization
